# Inguinal lymph node sample collected by minimally invasive sampler helps to accurately diagnose ASF in dead pigs without necropsy

**DOI:** 10.3389/fvets.2022.1000969

**Published:** 2022-09-28

**Authors:** Xiaowen Li, Yang Li, Mingyu Fan, Shiran Fan, Wenchao Gao, Jing Ren, Qingyuan Liu, Jingtao Li, Weisheng Wu, Junxian Li, Qiannan Yu, Xinglong Wang, Zhichun Yan

**Affiliations:** ^1^Shandong New Hope Liuhe Agriculture and Animal Husbandry Technology Co., Ltd., (NHLH Academy Swine Research), Dezhou, China; ^2^Xiajin New Hope Liuhe Agriculture and Animal Husbandry Co., Ltd., Dezhou, China; ^3^College of Veterinary Medicine, Northwest A&F University, Yangling, China; ^4^New Hope Liuhe Co., Ltd., Chengdu, China; ^5^Swine Health Data and Intelligent Monitoring Project Laboratory, Dezhou University, Dezhou, China

**Keywords:** African swine fever, inguinal lymph node, minimally invasive sampler, qPCR, diagnosis

## Abstract

African swine fever (ASF) is a highly contagious hemorrhagic and transboundary animal disease, and it threatens global food security. A full necropsy to harvest the sample matrices for diagnosis in the farm may lead to contamination of the premises and directly threaten to the herds. In the present study, we compared the ASFV loads of the common samples that can be collected without necropsy. The unmatched nasal, throat, rectal samples were randomly taken using cotton swabs, and inguinal lymph node samples were collected by the minimally invasive samplers from the dead pigs of an ASF field outbreak farm. The ASFV loads of the samples were detected by qPCR and the results suggested that the overall ASFV nucleic acids levels of inguinal lymph node samples were higher than the swabs. What's more, sets of matched nasal swabs, rectal swabs, throat swabs, inguinal lymph nodes, serums, spleens and lungs samples were collected from 15 dead ASFV naturally infected pigs. Similarly, the results showed that inguinal lymph node samples, together with serum, spleen and lungs samples, contained more ASFV nucleic acids than the swabs. Our findings demonstrated that the inguinal lymph node collected by minimally invasive sampler is an ideal tissue for diagnosing ASFV infection in dead pigs without necropsy.

## Introduction

African swine fever (ASF) is a devastating swine viral disease that is reportable to the World Organization for Animal Health (WOAH). It causes high fever, severe depression, ataxia and hemorrhages in domestic swine and results in a mortality rate approaching 100% (1). Since its emergence in Kenya in the 1920's, ASF remains endemic in sub-Saharan Africa, the Indian Ocean region and Eastern Europe (2–4). In 2007, ASF was confirmed in Georgia and Russia, posing the risk of further dissemination into neighboring countries (5). Since August 2018, ASF has spread to China, the world's largest pork producer and a leading pork importer (6). Ongoing outbreaks of ASF afflicted the livestock industry and wiped out 40% of the nation's pigs, leading to severe socioeconomic losses (7–9).

The causative agent of the disease, ASF virus (ASFV), belongs to the genus *Asfivirus* within the *Asfarviridae* family (10). ASFV is a complex enveloped virus containing a large double-stranded DNA genome that ranges in length from 170 to 193 kbp (11, 12). Although scientists worldwide have made great efforts to study the etiology and immunology of ASFV, no prophylactic vaccine or treatment option is widely accepted and used. Clinical diagnosis is impractical for the similar classic symptoms of ASF and classic swine fever, swine erysipelas and highly pathogenic porcine reproductive and respiratory syndrome. Laboratory diagnosis is indispensable to make a definite diagnosis for the dead ASF suspected pigs. WOAH recommends two types of laboratory diagnosis, including etiological diagnosis and serological diagnosis. To date, qPCR has been widely used to detect ASFV nucleic acids due to its simple, rapid, highly sensitive and specific features. Nevertheless, the quantity and quality of conventional samples, such as oral, nasal and rectal swabs vary from pig to pig. False-negative results pose a concerning threat to herds. High ASFV loads was found in organs, including brain, heart, spleen, tonsil, bone marrow, lung, liver, kidney and lymph node (10, 13–15). However, opening up the carcasses is time-consuming and skilled, which often leads to contamination of the premises and increases the risk of ASFV infection in the herds (16). Researchers reported that the average viral loads of lymph nodes were comparable with internal organs (14, 15). The purpose of this study was to compare the ASFV loads of inguinal lymph nodes and the common swabs that can be collected without a full necropsy.

## Method

**Figure 1:**
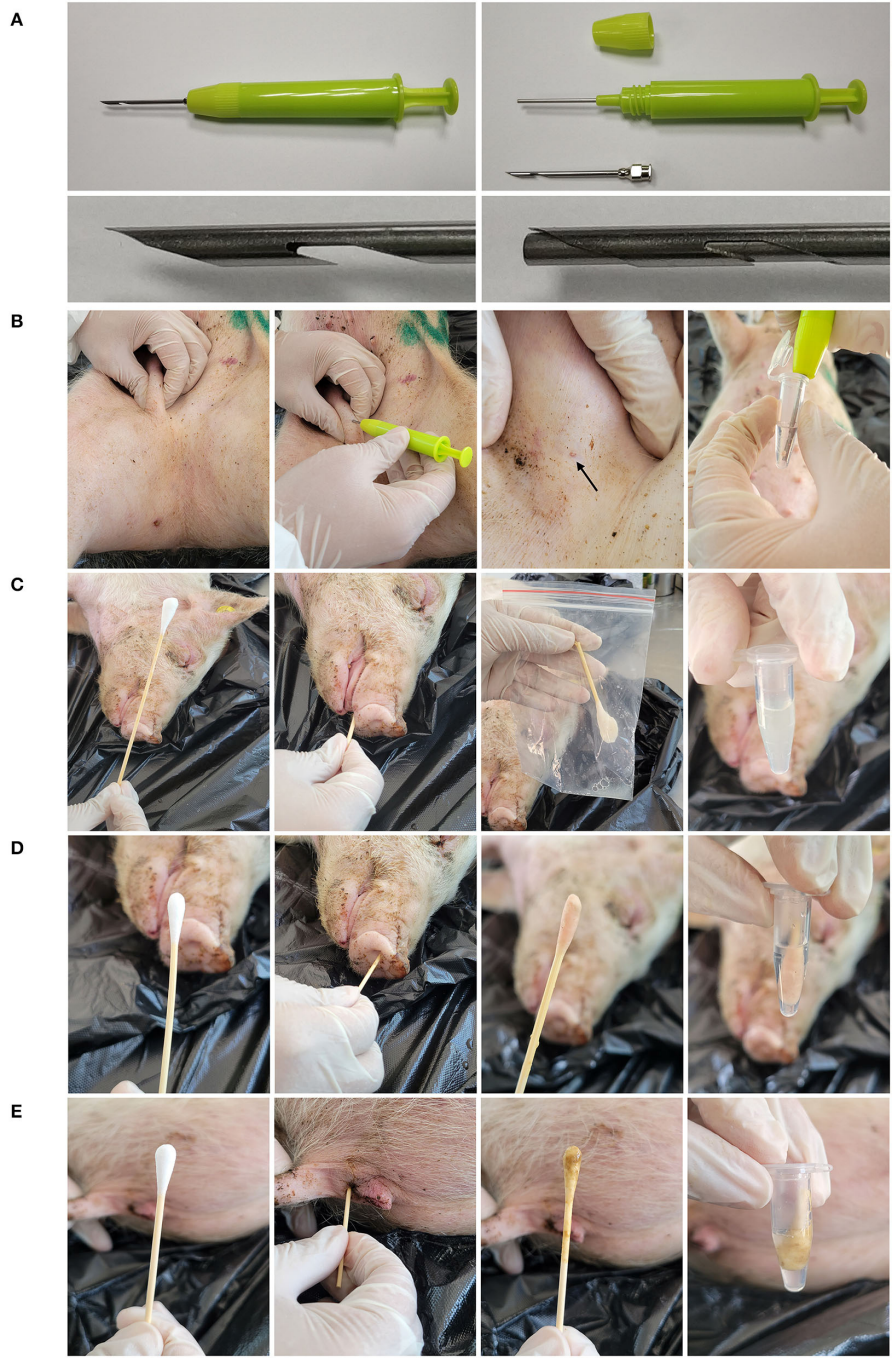
Sampling tools and methods. **(A)** The structure of minimally invasive sampler. The sample contains three parts: the needle, the body and the screw cap. The connection rod can insert into the needle to extrude the tissue. **(B)** Collect the inguinal lymph node sample with the minimally invasive sampler. First, fix the inguinal lymph node and puncture the skin with the sampler. Then pull out the sampler and extrude the tissue into 0.5 mL saline solution. **(C)** Collect the throat sample with a long swab. Insert the swab into the throat and moved back and forth for five times. The swab was then eluted in 1 mL saline solution in a sealing bag. The eluate was stored in the centrifuge tube. Collect the nasal swab sample **(D)** and rectal swab sample **(E)** with short swabs. The swabs were inserted into the nostril or anus and moved back and forth for five times. The swabs were then eluted in 0.5 mL saline solution.

This study was performed in line with the principles of the Declaration of Helsinki. Approval was granted by the Technology Ethics Committee of the Dezhou University (DZU/IACUC_2022-2-18-1). The unmatched inguinal lymph node samples, throat swabs, nasal swabs and rectal swabs were collected randomly from the dead-ASFV positive pigs of a naturally infected 1–2-year-old Landrace-Duroc-Large white crossbred swine herd in Xiajin County, Shandong Province, China (116°16′67″E, 37°13′17″N). For inguinal lymph node samples, the minimally invasive sampler was used which consists of a needle, a syringe, a handle and a connection rod inside the needle (Figure 1A). The needle contains a barb, which can remove the lymphoid tissue from the muscle. The connection rod can be inserted into the needle to extrude the tissue. As shown in Figure 1B, the skin was punctured vertically with the sampler to ensure that the barb entirely entered the tissue. The sampler was pulled out, and the handle was pressed to push the tissue out of the needle. The tissue was placed into a centrifuge tube and submerged in 0.5 mL saline solution. For throat swab sample, a long swab was inserted into the throat and moved back and forth for five times. The mucus was eluted with 1 mL saline solution in a sealing bag and the eluate was transferred into a centrifuge tube (Figure 1C). The nasal swabs (Figure 1D) and rectal swabs (Figure 1E) were collected using the short swabs and eluted with 0.5 mL saline solution. The matched inguinal lymph node samples, throat swabs, nasal swabs and rectal swabs were collected from 15 naturally infected dead-ASFV positive pigs using the same methods. Blood in thorax were centrifuged at 3,000 g for 2 min to separate serum. Sliced spleen and lung samples were collected from a full necropsy in a slaughterhouse.

To determine the viral loads in each sample, the following primers specific for ASFV B646L gene were designed based on the ASFV isolate Pig/HLJ/18 (GenBank: MK333180.1) (10) and used for qPCR: 5'-AAAATGATACGCAGCGAAC-3' (forward), 5'-TTGTTTACCAGCTGTTTGGAT-3' (reverse), 5'-FAM-TTCACAGCATTTTCCCGAGAACT-BHQ1-3' (probe). The qPCR was carried out in a Step One Plus instrument (ABI) with PerfectStart^®^ II Probe qPCR SuperMix (TransGen Biotech, China) according to the manufacturer's instructions. To process the samples, homogenates of lymph nodes, spleens and lungs were prepared using Precellys lysing kits with the Precellys tissue homogenizer (Bertin, France). Swab samples were oscillated and then centrifuged at 8,000 g for 2 min. The total DNA of each sample (200 μL) was extracted by Virus DNA Extraction Kit II (Geneaid, Taiwan) as described in the handbooks without modification. Five microliters of extracting solution was taken for qPCR detection. Results of qPCR were initially recorded as quantification cycle values.

Partial sequences of ASFV B646L were cloned into the pMD18-T vector (Takara) to construct the ASFV DNA standard plasmids (Supplementary material 1). Briefly, B646L gene partial sequences were amplified using the following primers: 5'-TACGAATTCGTTGGCCAGGAGGTATCGGT-3' (forward), 5'-AGAGGATCCAAGAGGGGGCTGATAGTATT-3' (reverse). Amplification was carried out using a pre-mixed PCR solution (P111-01, Vazyme) according to the instructions. As a standard procedure, 40 consecutive cycles of thermal denaturation at 95°C (15 s), primer annealing at 60°C (15 s) and primer extension at 72°C (30 s) were carried out in a PCR thermocycler (C1000, Bio-Rad). PCR products and pMD18-T plasmids were digested with same restriction enzymes (EcoR I and BamH I, Takara). Fragments were purified by agarose gel electrophoresis and linked by DNA ligases (C301-01, Vazyme) according to the standard procedure. Positive clones were screened out with Ampicillin culture dish and identified by sequencing. The standard curve was obtained using a known concentration of the recombinant T vector, which was serially diluted from 10^8^ to 10^3^ copies/μL. The detection limit of qPCR assay is 2.5 copies/μL of the ASFV genome. The sample CT values were converted to ASFV copy numbers based on the standard curve. GraphPad Prism v8.3.0 was used to make graphs and analyze the data by *t*-test.

## Results

**Figure 2:**
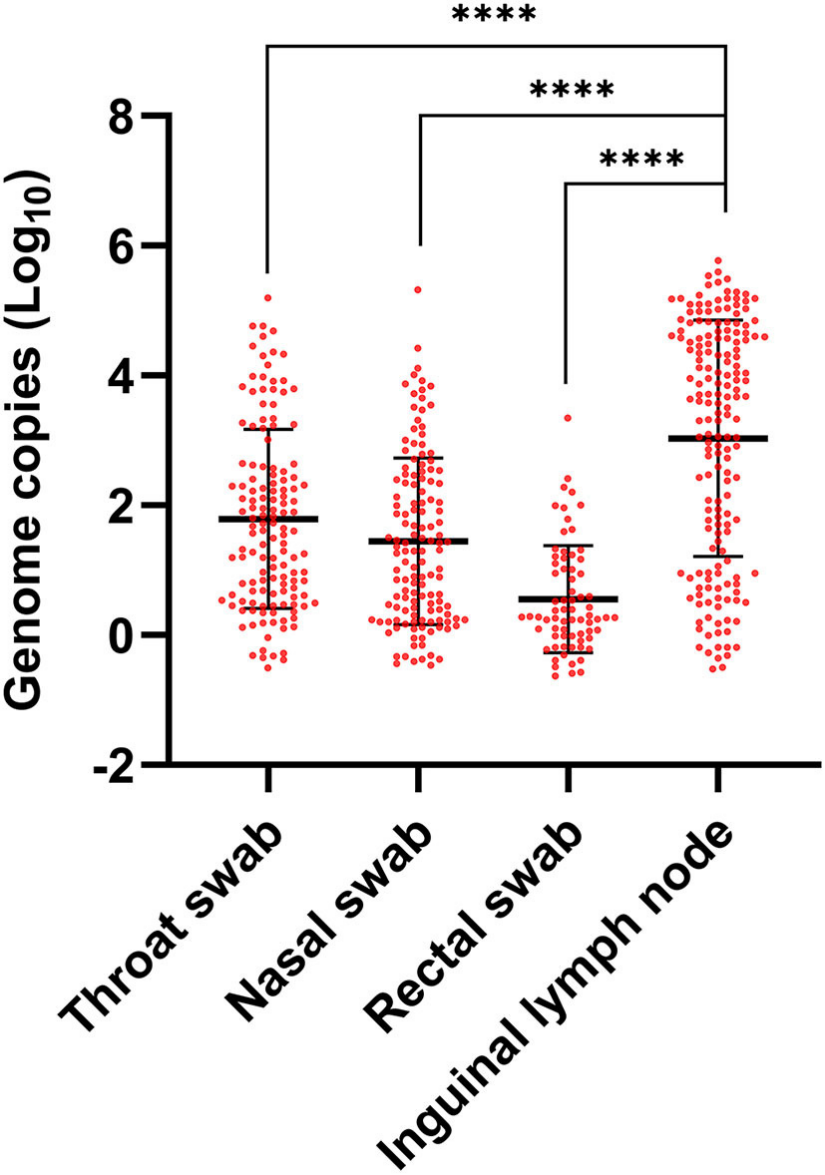
ASFV contents in lymph nodes and swabs. Throat, nasal and rectal samples were randomly collected by swabs and inguinal lymph nodes were collected by minimally invasive sampler from the dead pigs of an ASF field outbreak. ASFV contents were detected by qPCR using specific primers and probes targeting B646L gene. Individual CT values were converted to viral genomic log_10_ copy numbers based on an established standard curve. The difference analysis was carried out by GraphPad Prism v8.3.0 using unpaired *t*-test. ****p value < 0.0001.

A total of 175 lymph node samples, 137 throat swab, 134 nasal swab, 71 rectal swab samples randomly collected from the ASFV infected dead pigs were tested to determine the copy numbers of ASFV by qPCR. The overall highest copy numbers of ASFV were found in the inguinal lymph node samples (*p* < 0.0001, unpaired *t*-test) compared with the swab samples, and the lowest copy numbers were found in the rectal samples (Figure 2). The nasal and throat swab samples had almost the same ASFV copies, which were relatively lower than the lymph node samples and higher than the rectal samples. These data suggested that the overall ASFV loads of inguinal lymph node were higher than the unmatched swab samples in the field.

**Figure 3:**
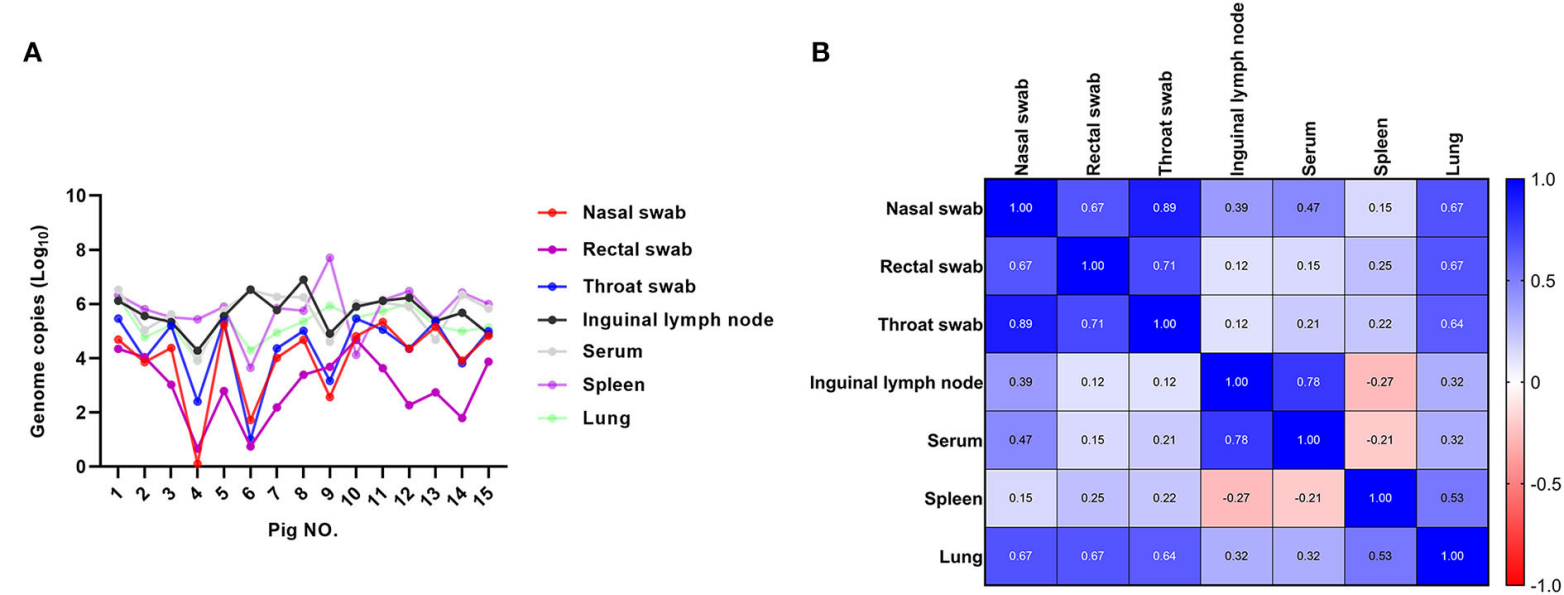
The comparison of ASFV contents of different sample types from the single pig. Sets of matched nasal swabs, rectal swabs, throat swabs, inguinal lymph nodes, serums, spleens and lungs of 15 pigs were collected and the ASFV contents were detected by qPCR. **(A)** Individual CT values were converted to viral genomic log_10_ copy numbers. **(B)** The correlation values of ASFV genome copies between different types of samples were calculated using GraphPad Prism v8.3.0. Deeper blue means there are stronger positive correlation between the viral contents of different kinds of samples. Similarly, red color indicates the negative correlation.

To compare the ASFV loads in different samples more directly, we collected the throat swab, nasal swab, rectal swab samples and inguinal lymph node sample from the single dead pig (*n* = 15). Pigs were then dissected to collect the matched serum, lung and spleen samples. All samples were tested for ASFV contents by qPCR. As is shown in Figure 3A, viral loads of lymph node samples were comparable with serums, spleens and lungs. All of the 15 inguinal lymph node samples had higher ASFV copies than the swab samples. Over all, the ASFV copies of the throat swabs were parallel with the nasal swabs, and 12 of 15 rectal swabs had the lowest ASFV copies. These data demonstrated that inguinal lymph nodes contain higher ASFV loads than the swab samples.

When the ASFV genome copy numbers in the inguinal lymph node samples were compared with those of the throat swabs, nasal swabs and rectal swabs, there was a weakly positive correlation (*r* = 0.12, 0.39, and 0.12, respectively). Though all of lymph nodes, spleens and lungs samples contain high ASFV copies, there was no strong correlativity. However, strong positive correlation was found between the viral contents of serum and inguinal lymph node samples (*r* = 0.78). Interestingly, strong positive correlation (*r* = 0.89) also existed between the respiratory and alimentary samples, including rectal swabs, throat swabs, nasal swabs and lungs (Figure 3B). These results indicated that even though ASFV genomic copy numbers of throat swabs, nasal swabs and rectal swabs were relative, but they were less than the inguinal lymph node samples as well as the serum and internal organs.

## Discussion

ASF is an acute viral infection that has a major impact on global pig production. The availability of effective and safe ASFV vaccines would support and enforce control–eradication strategies. Therefore, work leading to the rational development of protective ASF vaccines is a high priority (17–20). It was recently reported that a promising recombinant vaccine candidate, ASFV-G-ΔI177L, was developed by deleting the I177L gene from the genome of the highly virulent ASFV strain Georgia (21–24). However, the presence of efficacy, residual virulence and the possibility of reversion to virulence must be evaluated in the field, which will need much further research before commercialization. Due to the lack of vaccine or effective treatments, prevention, control and eradication measures are mainly based on enhanced biosecurity and early detection by efficient laboratory diagnosis (18).

qPCR assays have many advantages compared with immunology methods, such as simple operation processes, high sensitivity, high specificity and excellent repeatability. Thus, qPCR is the most commonly used laboratory test method to diagnose ASFV at present. The low viral load, of course, also means infection, as long as the test results are indeed positive. However, in fact, weakly positive results always puzzle the experimenter because of the possible reagents contamination and aerosol contamination. The most challenging thing of qPCR is how to get the samples with higher viral loads. The internal organs such as spleen, lymph nodes, bone marrow, lung, tonsil and kidney contain high viral loads and are WOAH-recommended samples for ASF dead pigs, however, opening up the carcass is inevitable and risky (25). A robust viremia generally occurs within 2 days post ASFV infection and serum was an usual sample to diagnose the disease (26, 27). However, blood clotting happens within hours after the death, which means serum is always unavailable in dead pigs. ASFV spreads through the lymph and blood to secondary lymphoid organs within 2–3 days, thus the lymphoid grans were thought be an ideal tissue type for early detection of ASFV (28). It's interesting to note that viral content of inguinal lymph node sample has strong positive correlation with that of serum (Figure 3B), suggesting that inguinal lymph node sample can replace serum to detect ASFV in dead pigs. Actually, lymph nodes were used for viral loads measurements in many studies (29–31). Among the superficial lymph nodes, inguinal lymph node is the largest and the most accessible one. ASFV genome copy numbers in superficial inguinal lymph nodes highly correlate with those in the spleen and its sensitivity to diagnose ASF oro-nasally infection is 100% (16).

For the absolute avoidance of environmental contamination, the minimally invasive sampler instead of surgical operation was utilized to puncture the skin in a minimally invasive manner to obtain lymph node tissue in the present study. As a result, 10 of 15 inguinal lymph node samples showed much higher ASFV genomic copy numbers than the swabs, while the other five contains similar but no less viral loads compared with the throat swabs and nasal swabs. This possibly due to the sampling errors caused by the sampling position deviation, reminding us that the operating personnel should be well-trained to puncture the lymph node accurately with the minimally invasive sampler.

The needle of the minimally invasive sampler costs a bit more than a normal steel syringe needle. The sampler body and the needle can be recycled after cleaning and disinfection, which helps to keep costs down. What's more, the sampler helps to increase the confirmed diagnostic rate and avoid the risk of ASFV spread, which may sometimes save the whole herd in the ASFV epidemic area. We believe that the costs of minimally invasive sampler are acceptable to most ASF-disturbed breeders.

In conclusion, the ASFV viral loads was proved to be higher in the inguinal lymph nodes than the throat, nasal and rectal swabs samples. Considering the threaten of ASFV contamination to the herds, the lymph node samples collected by minimally invasive sampler can be an optional alternative of a full necropsy or a surgical operation to diagnose ASF in dead pigs.

## Data availability statement

The datasets generated during the current study are available from the corresponding author on reasonable request.

## Ethics statement

The animal study was reviewed and approved by Technology Ethics Committee of the Dezhou University. Written informed consent was obtained from the owners for the participation of their animals in this study.

## Author contributions

ZY and XL contributed to the study conception and design. SF, WG, QL, JiL, WW, JuL, and QY performed the material preparation and data collection. JR, MF, XW, and YL performed the analysis. YL and XW wrote the draft of the manuscript. All authors read and approved the final manuscript.

## Funding

This work was supported by the Integration and Demonstration of Comprehensive Prevention and Control of ASFV by the Ministry of Science and Technology of the People's Republic of China (No. 2018YFC0840405), the Scientific and Technological Innovation 2030 Program of Ministry of Science and Technology of the People's Republic of China (No. 2021ZD0113803), and the National Natural Science Foundation of China (No. 31701424).

## Conflict of interest

Authors XL, YL, MF, SF, WG, QL, JiL, WW, JuL, QY, and ZY were employed by New Hope Liuhe Co., Ltd., China, Xiajin New Hope Liuhe Agriculture and Animal Husbandry Co., Ltd., Dezhou, China, and/or Shandong New Hope Liuhe Agriculture and Animal Husbandry Technology Co., Ltd., China. The remaining authors declare that the research was conducted in the absence of any commercial or financial relationships that could be construed as a potential conflict of interest.

## Publisher's note

All claims expressed in this article are solely those of the authors and do not necessarily represent those of their affiliated organizations, or those of the publisher, the editors and the reviewers. Any product that may be evaluated in this article, or claim that may be made by its manufacturer, is not guaranteed or endorsed by the publisher.
