# Supplementary material for: Inguinal lymph node sample collected by minimally invasive sampler helps to accurately diagnose ASF in dead pigs without necropsy
Source: Front Vet Sci. 2022 Sep 28;9:1000969. doi: 10.3389/fvets.2022.1000969 (PMC9554536; doi:10.3389/fvets.2022.1000969)
Supplement: Supplementary file 1 [file Data_Sheet_1.DOCX]

>Cloned B646L gene partial sequences

GTTGGCCAGGAGGTATCGGTGGAGGGAACCAGTGGCCCTCTCCTATGCAACATTCATGATTTGCACAAGCCGCACCAAAGCAAACCTATTCTTACCGATGAAAATGATACGCAGCGAACGTGTAGCCATACCAACCCGAAATTTCTTTCACAGCATTTTCCCGAGAACTCTCACAATATCCAAACAGCAGGTAAACAAGATATTACTCCTATCACGGACGCAACGTATCTGGACATAAGACGTAATGTTCATTACAGCTGTAATGGACCTCAAACCCCTAAATACTATCAGCCCCCTCTT
